# Interaction between fire and fragmentation in the successional stages of coastal dune grasslands of the southern Pampas, Argentina

**DOI:** 10.1038/s41598-019-51595-x

**Published:** 2019-10-22

**Authors:** Alejandra L. Yezzi, Ana J. Nebbia, Sergio M. Zalba

**Affiliations:** GEKKO - Grupo de Estudios en Conservación y Manejo, Departamento de Biología, Bioquímica y Farmacia, Universidad Nacional del Sur, San Juan 670 (8000), Bahía Blanca, Argentina

**Keywords:** Conservation biology, Plant ecology, Invasive species

## Abstract

Vegetation’s increased vulnerability to extrinsic disturbances is an important but less studied effect of natural habitat fragmentation. Fire is part of the evolutionary history of grassland ecosystems, but fragmentation by forest plantations can alter the fire regime and influence their resilience. This study compares the successional trajectories after fire in continuous and fragmented grassland in terms of composition and abundance of plant species. Grassland fragments of varying sizes (0.1 to 2.5 ha) surrounded by a forest matrix and grassland controls of an equivalent area in adjacent, non-fragmented sites were selected. Fire was associated with an increase in the abundance of exotic plants in the fragmented grassland whereas the continuous grasslands were much more resistant to invasion. These differences in the species composition between fragments and continuous areas, which were limited to the smaller areas before the fire, were observed one year after the fire throughout the range of sizes analyzed. These results show the impact of fragmentation on grassland resilience and how the effects of this process become evident even months after a disturbance, highlighting the synergistic effect of habitat fragmentation and biological invasions, two factors identified as the main forces of biodiversity erosion.

## Introduction

The fragmentation of natural habitats is one of the main processes responsible for the global crisis of biodiversity loss and the degradation of ecosystems around the world^1,2^, and consists of the division of a continuous area of natural habitat into two or more fragments surrounded by a modified landscape matrix^3^. The isolation of the fragments generated and the influence of the matrix from the resulting edges, increase the vulnerability of vegetation to extrinsic disturbances such as fire^4^. The importance of this greater vulnerability has been less recognized than other aspects of habitat degradation; however, it has significant implications for the long-term viability of fragmented habitats^5^. Wilson *et al*.^2^ argue that habitat fragmentation can alter successional trajectories and suggest that the intensity of these changes will depend on the intensity of the disturbance and the size of the area that is being disturbed. However, synergies like the interaction between fragmentation and an extrinsic disturbance, such as fire, need to be further studied^1^.

Grassland is recognized as the terrestrial biomes with biodiversity and ecosystem services that are most at risk worldwide due to the great disparity between the rate of habitat loss and the degree of effective protection^6,7^. Most studies on fragmentation have focused on the replacement of areas occupied by natural forests by fragments surrounded by pastures and crops^4,8,9^, whereas much less is known about the opposite change, i.e. the establishment of forest plantations in grassland areas^10,11^. In particular, the grasslands of southeastern South America have undergone a process of transformation and fragmentation due to the advance of various anthropogenic interventions^12^, and grasslands in good conservation status are restricted to habitats where the agricultural edge has not been able to advance, such as in rocky outcrops, wetlands, and coastal dunes^13,14^. Beyond the limits imposed by agriculture on the nutrient-poor soils of coastal dune habitats, the plant communities of these habitats face the challenge of urbanization and afforestation with conifers and other exotic tree species^12^.

Fire has been one of the main selective forces in grassland ecosystems; native species have evolved with this natural disturbance and therefore acquired adaptations that allow them to survive^15,16^. Fire is an important factor in the ecological successional of plant communities, but its effects on the species composition of vegetation and ecosystem functions vary with its intensity, frequency, and duration^17,18^. Natural fire regimes are different in different biomes and between different habitats, since the structure of the preexisting vegetation conditions fire intensity^19,20^. Afforestation in prairie habitats generates particularly significant changes in the characteristics of fires. This is because the temperature and the severity of the fire increase with the amount of easily combusted material accumulated, whereas the burnt grassland areas experience relatively low temperatures that do not completely eliminate the vegetation cover and do not affect the organic carbon reservoirs in the soil. Fragmentation by forest plantations can alter the traditional fire regime in grassland, increasing its negative effects on the vegetation and soil. Many disturbances of human origin modify some properties of the system that deeply condition their successional response, altering species composition by decreasing the abundance of native plants and creating opportunities for exotic plants to invade^15,16,21,22^. Fragmentation by forest plantations is likely to alter fire regimes and also to influence the resilience of fragmented grassland by changing species composition and its response after the disturbance^20^.

In Argentina, the coastal dunes of the Pampas are better conserved comparatively than the rest of the grasslands, but they also present threats derived from anthropogenic activities that result in habitat loss and fragmentation, mainly through the establishment and expansion of urban nuclei and afforestation with exotic species^12,23,24^. Afforestation fragments the coastal ecosystem, changing the species composition of the vegetation and increasing the number of exotic plants, mainly in remnants of smaller size^11^. This study focuses on the interaction between fragmentation (a result of human activities) and fire (a natural disturbance) in grasslands growing on sand dunes on the coast of Buenos Aires province (Argentina), surrounded by a plantation of *Pinus pinaster* (maritime pine), a species with invasive behaviour^25–27^. In this paper, we analyze the effects of this synergy by evaluating the recovery processes after the fire in continuous and fragmented grassland^28^ in terms of composition and abundance of plant species. We propose that fragmentation will affect the successional trajectory of the burnt habitats, with increases in the abundance of exotic species in the fragments immersed in the forest matrix with respect to the continuous grassland, and that this difference will be more significant in the smaller fragments.

## Materials and Methods

### Study area

The coastal dunes of the extreme south of the Pampas^29^ occupy the southwest of Buenos Aires province, Argentina, on a strip along the Atlantic coast that extends between approximately 38°55′46″S – 60°30′32″W and 39°00′12″S – 61°33′12″W. This coastal strip varies between two and seven kilometers in width, including the beaches, bare dunes, dunes covered with vegetation, and depressions between the dunes with small lagoons associated to humid grassland. The climate is temperate, being influenced by the proximity to the sea^30^. Communities of the species *Hyalis argentea*, *Panicum urvilleanum*, *Thelesperma megapotamicum* and *Oenothera* sp. dominate the dunes^31^. Pampas grass (*Cortaderia selloana*), rushes (*Juncus acutus*), sedges (*Schoenoplectus americanus*) and bulrushes (*Typha latifolia*) are found in the depressions between the dunes^32,33^.

The natural landscape is well-preserved^34^, free of crops and subject to extensive livestock production. The area includes tourist localities associated with plantations of eucalyptus (*Eucalyptus* spp.) and pines (mainly *Pinus pinaster* and *P*. *radiata*). The latter has the ability to expand spontaneously over dune habitats^35^. This study was carried out in a set of natural grassland remnants of different sizes surrounded by a plantation of maritime pine (*P*. *pinaster*; 38°55′S – 60°33′O) covering 30 ha, and in adjacent continuous grassland. Both the plantation and the continuous grassland were completely burnt by a wildfire on January 6th, 2014.

### Study design and field sampling

Remnants of natural vegetation surrounded by the pine plantation were identified from a satellite image of the site taken from Google Earth and dated December 14th, 2006. Their surface area was measured using Google Earth Pro. The fragments were identified in the field, confirming that there had been no changes in their surface area and shape since the capture of the satellite images. Six grassland fragments of up to 0.1 ha were selected, three from 0.1 to 0.5 ha, and six from 0.5 to 2.5 ha, totalling 15 sample units. Fifteen continuous grassland controls without afforestation of equivalent surface areas were selected in neighbouring areas.

The data used for this study were taken before the fire occurred, and then three and fifteen months afterwards. In each case, we estimated the percentage cover of all plant species present and the cover of bare ground following the method of *relevés* of Braun Blanquet^36^. Plants were identified to the species and/or genus level^37^ and were classified as native or exotic. The Catalog of Vascular Plants of the Southern Cone^38^ was used for nomenclature.

### Data analysis

Ternary diagrams were used to observe the progression of changes in the cover of native and exotic plants and of bare ground in fragments of different sizes and in the continuous grassland controls before the fire, and three and fifteen months after the fire.

The Kruskal-Wallis test with multiple comparisons as post hoc tests^39^ were applied to analyse the effects of the successional stage (before the fire, and three and fifteen months afterwards), the context (fragmented grassland or continuous grassland), and the size of the areas (small, intermediate, or large), on the cover of native and exotic plants and of bare ground. The normality of the data was evaluated for the study of residuals and of normal probability plots. The Levene test was used to verify homogeneity of variances.

Species composition of the sampling sites was compared by means of a Principal Component Analysis (PCA) based on the covariance matrix of the species cover data with the objective of identifying sets of species associated with the size and context of the samples, and with the different successional stages related to fire. The data were transformed using the arc sine of the square root to correct the distortion of the Euclidean distance in its spatial representation^40^. To maintain an appropriate balance between the number of samples and variables (coverage by species) the plants present in less than 33% of the samples (75 species) was excluded from the analysis. All analyses and graphs were performed using the Infostat and XLSTAT statistical packages.

## Results

**Figure 1:**
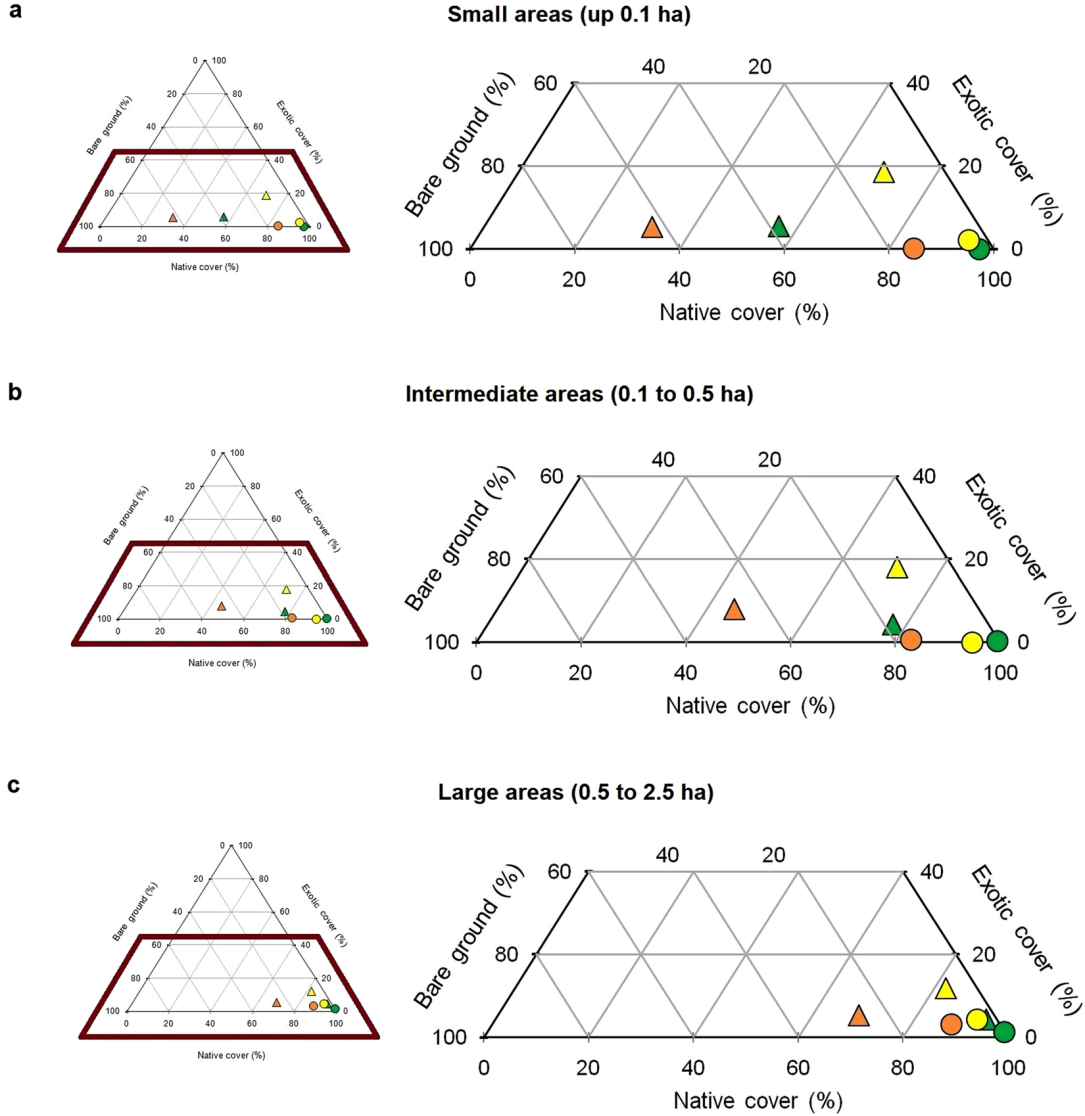
Changes in the cover of native and exotic plants, and of bare ground in grassland fragments surrounded by a pine plantation (triangles) and continuous grassland controls of equivalent area (circles) in small (**a**), intermediate (**b**) and large (**c**) areas. The colors represent successional stages: before the fire (green), and three (orange) and fifteen (yellow) months after the fire.

The after-fire successional trajectories of the grassland fragments surrounded by pine plantations and of the continuous grassland controls were qualitatively similar with respect to the cover of the native and exotic plants and of bare ground, regardless of the size of the plots. There was a decrease in the cover of native species three months after the fire and an increase after fifteen months. The percentage of bare ground showed the opposite behaviour, whereas the cover of exotic species was constant throughout the entire sampling period, except in the case of the fragments surrounded by the forest matrix, where it increased fifteen months after the fire. The changes described were much more noticeable in the fragments than in the continuous grassland controls. The greatest differences between the fragments and controls were observed in the smaller areas (Fig. 1).

**Figure 2:**
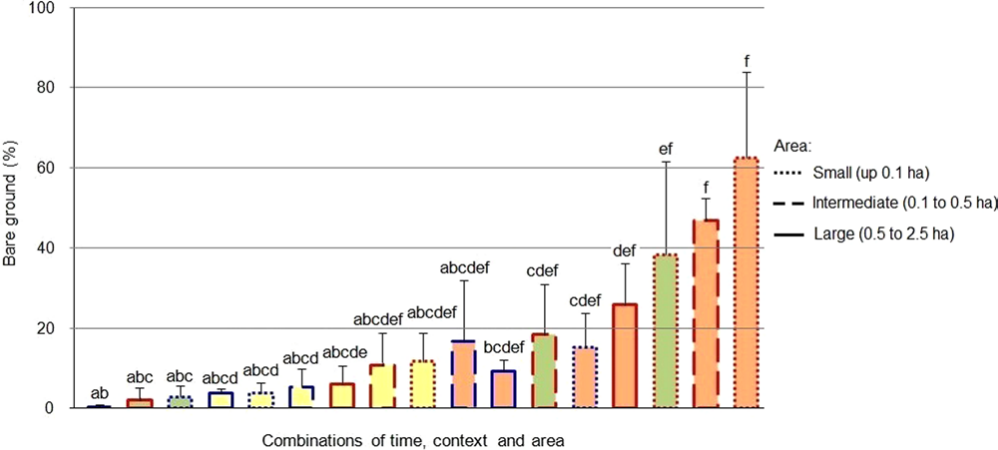
Comparison between the bare ground in grassland fragments surrounded by a pine plantation (red edge) and in continuous grassland controls (edge blue) of different surface areas, at different successional stages in relation fire (green = before; orange = three months later; yellow = fifteen months later). Different letters indicate significant differences (P < 0.05); bars represent the standard error.

**Figure 3:**
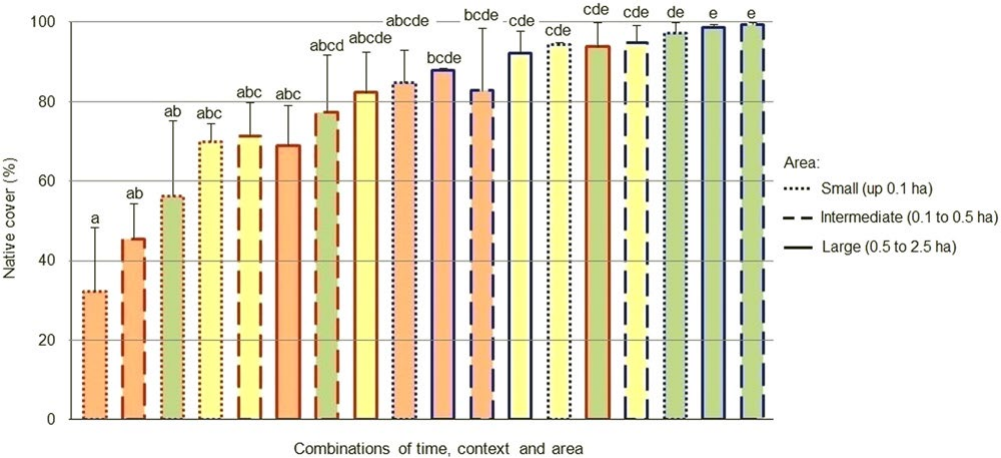
Comparison between the cover of native plants in grassland fragments surrounded by a pine plantation (red edge) and in continuous grassland controls (edge blue) of different surface areas, at different successional stages in relation fire (green = before; orange = three months later; yellow = fifteen months later). Different letters indicate significant differences (P < 0.05); bars represent the standard error.

**Figure 4:**
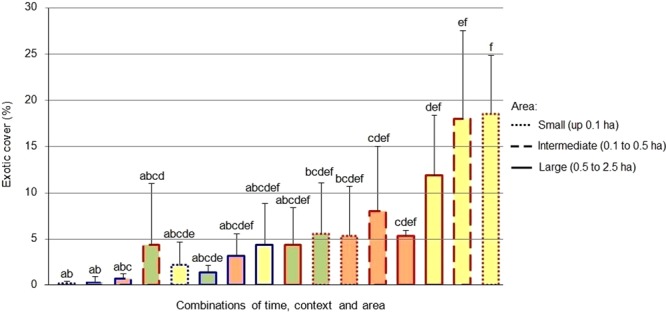
Comparison between the cover of exotic plants in grassland fragments surrounded by a pine plantation (red edge) and in continuous grassland controls (edge blue) of different surface areas, at different successional stages in relation fire (green = before; orange = three months later; yellow = fifteen months later). Different letters indicate significant differences (P < 0.05); bars represent the standard error.

An effect of the context, the area and the successional stage in relation to the fire was observed for both the bare ground and the vegetation cover (H = 40.97, P = 0.0009 for bare soil; H = 43.37, P = 0.0004 for native; H = 39.14, P = 0.0012 for exotic). Before the fire, the cover of bare ground was greater in the small and intermediate fragments with respect to the controls, but showed no differences in the larger areas. In the continuous grassland the bare ground cover did not vary with area, whereas in the fragmented grassland it decreased in the larger areas. No significant differences were observed between fragments and controls for any of the sizes analyzed three and fifteen months after the fire. In both contexts (fragmented grassland and continuous grassland) the proportion of bare soil was greater three months after the fire compared to the pre-fire stage (Fig. 2). In the pre-fire stage, the cover of native plants was significantly higher in the continuous grassland controls compared to the fragments surrounded by afforestation for the small and intermediate areas. The cover native plants remained unchanged with the increase in area in the controls, but increased in the larger fragments. In the stages after the fire, no significant differences were observed in the cover of native plants between fragments and controls for any of the sizes analyzed, nor between the different areas within each context (Fig. 3). The cover of exotic plants was significantly higher in the fragments compared to the controls in the small areas before the fire, remaining unchanged with the increase in area in both contexts (fragmented grassland and continuous grassland). Three months after the fire, no significant differences were observed, while fifteen months later the coverage of exotic plants was significantly higher in the fragments compared to the controls in the small and intermediate areas. The intermediate fragments showed a greater coverage with respect to the pre-fire stage (Fig. 4). Fifteen months after the fire, 24% of the coverage of exotic species in the large fragments corresponded to the pines that advanced from the matrix, representing a six-fold increase over the pine cover before the fire (4%). Pine coverage in the same time interval increased from 0% to 22% in the case of intermediate fragments and from 0% to 23% in small fragments.

**Figure 5:**
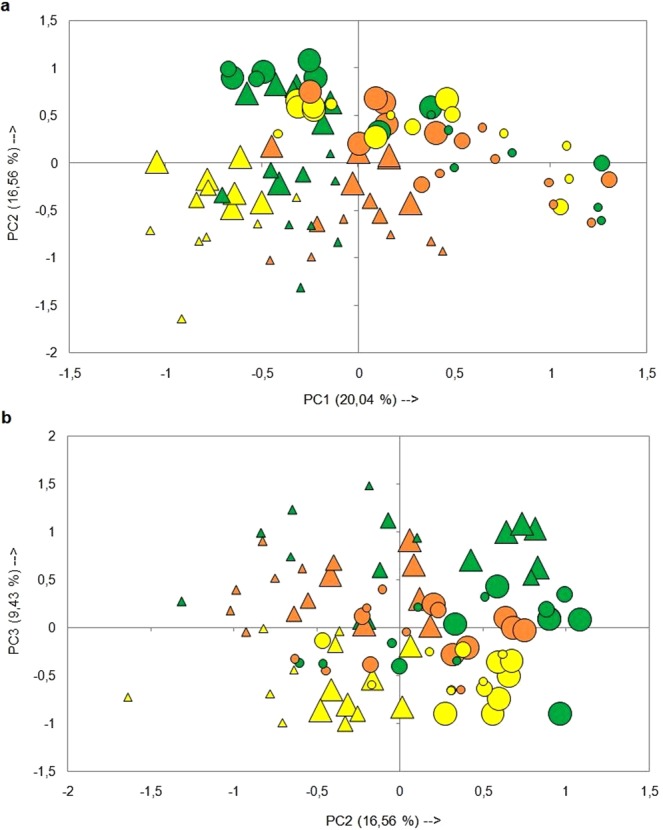
Principal Component Analysis (PCA) of sampling units of different size and at different successional stages in relation to fire located in sectors of continuous grassland (circles) and surrounded by a pine plantation (triangles). (**a**) PC1 and PC2; (**b**) PC2 and PC3. Symbols of different sizes represent sample units of different area (small = up 0.1 ha; intermediate = 0.1 to 0.5 ha; large = 0.5 to 2.5 ha) and colors represent successional stages: before the fire (green), and three (orange) and fifteen months (yellow) after the fire.

The Principal Components Analysis resulted in a grouping of the samples according to the context (fragmented grassland and continuous grassland), the size of the areas, and successional stages with respect to fire. The first component (20.04% total variance) separated the smaller fragments surrounded by the forest plantation with respect to the smaller continuous grassland controls (Fig. 5a). The samples corresponding to the fragments formed a group on the left end of the component characterized by a greater abundance of exotic species: *Senecio madagascariensis*, *Hypochaeris radicata*, *Acacia longifolia* and *Pinus pinaster*, with negative correlations with the first component. At the other end of PC1, the plots corresponding to the controls formed a second group, associated with high coverage values of grasses and other native species typical of the Pampas grassland: *Aristida spegazzinii*, *Panicum urvilleanum*, *Schizachyrium plumigerum*, *Margyricarpus pinnatus* and *Discaria americana* (Table 1, Fig. 5a). The second component (16.56% total variance) separated smaller fragments belonging to the whole range of successional stages considered (grouped on the left) from the larger fragments before the fire and the controls over the entire period of time (concentrated on the right). The same component also separated fragments fifteen months after fire (on the left) from controls at the same sampling time (on the right), throughout the range of sizes analyzed (Fig. 5b). Samples on the left end of PC2 were associated with high coverage values of the exotic species *Hypochaeris radicata* and *Pinus pinaster*, while on the other extreme of the same axis two native species, *Imperata brasiliensis* and *Cortaderia selloana*, were responsible of the maximum loads (Table 1, Fig. 5b). The third component (9.43% total variance) separated the samples mainly as a function of their location in the successional trajectory in relation to fire (Fig. 5b). The plots surveyed before and three months after the fire formed a group on the positive side of the axis and were characterized by a greater abundance of two native species associated with disturbances: *Ambrosia tenuifolia* and *Tessaria absinthioides*, both showing positive correlations with the third component. At the other end of PC3, the plots sampled fifteen months after the fire formed a group associated with high coverage values of two native plants: *Schizachyrium plumigerum* and *Schoenoplectus americanus* (Table 1, Fig. 5b).

**Table 1:** Correlation coefficients between the abundance of plant species and the first three components of the PCA (PC1, PC2 and P3).

Species	PC1	PC2	PC3
Acacia longifolia*	−0.467	−0.426	−0.247
*Achyrocline satureioides*	−0.113	0.317	−0.246
*Ambrosia tenuifolia*	−0.013	0.477	0.537
*Aristida spegazzinii*	0.680	0.055	−0.347
*Baccharis genistifolia*	0.156	0.130	−0.079
*Baccharis glutinosa*	−0.317	0.296	−0.295
*Cenchrus longispinus*	0.086	−0.065	−0.123
Cirsium vulgare*	−0.349	0.013	0.081
*Conyza bonariensis*	−0.397	−0.262	−0.372
*Cortaderia selloana*	−0.578	0.561	−0.202
*Cyperus reflexus*	−0.405	−0.220	−0.042
*Discaria americana*	0.472	0.125	−0.196
*Eragrostis airoides*	−0.371	0.325	0.020
Eragrostis curvula*	−0.164	−0.189	0.160
*Hydrocotyle bonariensis*	0.032	−0.433	0.163
Hypochaeris radicata*	−0.480	−0.487	−0.145
*Imperata brasiliensis*	−0.239	0.786	−0.044
*Juncus acutus*	−0.656	0.300	0.074
*Juncus imbricatus*	−0.066	0.135	0.161
*Margyricarpus pinnatus*	0.498	0.127	−0.018
Melilotus albus*	−0.178	0.243	−0.258
*Oenothera mollissima*	−0.086	0.067	0.018
*Panicum urvilleanum*	0.634	0.317	−0.270
Pinus pinaster*	−0.450	−0.445	−0.253
*Pseudognaphalium leucopeplum*	−0.124	−0.107	−0.195
*Schizachyrium plumigerum*	0.531	0.239	−0.495
*Schoenoplectus americanus*	−0.600	−0.239	−0.502
*Senecio filaginoides*	0.309	0.084	−0.038
Senecio madagascariensis*	−0.527	−0.048	−0.184
*Solidago chilensis*	0.175	0.297	−0.300
*Tessaria absinthioides*	0.206	0.083	0.422

^*^Exotic species.

## Discussion

Since the beginning of the 20th century, it has been suggested that habitat fragmentation may alter the capacity of biodiversity to recover from additional disturbances^41^. Different authors have argued that ecosystem fragmentation can modify successional trajectories, resulting in small fragments being affected to a greater extent than large fragments^2,42^. Our study represents a contribution to the study of the effects of fragmentation on the successional response of natural grasslands affected by fire. Evidence is provided on how the recovery of grasslands growing on sand dunes after a fire varies according to whether they are part of a continuous area or fragmented and immersed in a forest matrix, and, according to the size of the fragments, on the increase in the susceptibility of the fragmented areas to the invasion by exotic species, and on the reduction of their resilience after fires.

Our results show that the total vegetation cover and bare ground cover followed similar trajectories in grassland fragments and in continuous grassland controls after the fire, and with respect to their pre-fire characteristics. As expected, the vegetation gave way to bare ground following the fire, reversing this trend fifteen months later. However, while these changes were barely significant in the continuous grassland, they were much more noticeable in the fragments surrounded by the forest plantations, particularly in the smaller areas, which at least shows a delay in the recovery of the vegetation cover in response to fragmentation. The interaction between fire, fragmentation, and abundance of exotic plants is of particular interest. For several decades the relationship between invasion by exotic plants and disturbances has been highlighted^43,44^, in particular, with respect to the effects of changes in the nature, intensity, and frequency of the disturbances on the success of the invaders^45,46^. Similarly, it is recognized that the fragmentation of grasslands generates changes in the floristic composition of the community, favouring the entry of exotic species, which typically behave opportunistically^11,47–49^. The fire is a central natural component in the structuring of grassland vegetation communities^17,18^. However, changes in the composition of plant communities will respond to the fragmentation process and the alteration of the disturbance characteristics according to the matrix that surrounds the fragments. It is expected that this response affects the succession of vegetation in fragmented grasslands and eventually favouring the invasion by exotic species. Our results show that the cover of exotic plants is greater in fragmented grasslands surrounded by the forest matrix with respect to continuous grasslands, and the differences observed only in small areas before the fire, also extend to intermediate sizes fifteen months after the disturbance. In particular, our results document the encroachment of the pines into the remnant grassland fragments from the forest matrix in response to the fire. The pines typically release large quantities of seeds after a fire and the environmental conditions post fire favor their germination and establishment^50,51^. The fires in these types of grassland surrounded by pine plantations probably resulted in subsequent habitat losses and the intensification of the fragmentation process. This increase in the cover of exotic plants in fragmented habitats fifteen months after the disturbance contrasts with the constancy in their relative importance throughout the successional process for the plots surrounded by continuous grassland. The intact grasslands thus appear to be more resistant to invasion, as had been reported for similar habitats affected by fire in South Africa^22^, whereas the fragmentation of grasslands growing on sand dunes in the study area seems to increase their vulnerability to invasions. This increase in invasiveness could be due both to a reduction in fragment resistance associated with previous alterations in its species composition, and to a higher pressure of propagules related to its proximity to the forest matrix.

Changes in species composition along the successional trajectory also provide evidence on the effects of fragmentation. The samples of intact grassland showed a marked coincidence in the dominant species before the fire and fifteen months later, evidence of resilience that was much less obvious in the fragments surrounded by the forest plantation. Whereas before the fire event only the smallest fragments showed a species composition different from that of the continuous grassland controls of equivalent areas, these differences between habitats became widespread throughout the whole size range (small, intermediate, and large areas) one year after the fire.

The results obtained have at least two fundamental implications: they reinforce the evidence of the effect of fragmentation on grassland resilience, and they show how the effects of the fragmentation process, which can go unnoticed in certain successional stages, become evident after a disturbance. It is also important to note that the forest matrix could have affected the recovery capacity of grassland fragments not only in terms of the fragmentation process itself, but also due to the characteristics of a fire that affects a pine plantation. It is expected that the temperatures reached within the fragmented have exceeded those typical of grassland fires^52^, particularly affecting small fragments and thus confounding the particular effect of fragmentation.

Our study demonstrates the synergistic effects of the two main causes of biodiversity erosion in grassland remnants of high conservation value: habitat loss and fragmentation, and biological invasions^53^. It is also important to highlight the possible feedback between disturbance and habitat loss, as some of the invasive species recorded in this study, such as pines and acacias, have evolved in fire-adapted ecosystems. Changes in the dynamics of fires may favour their expansion, further tilting the balance in their advantage in this scenario of fragmentation and habitat loss^15,54,55^.
